# Endothelin-1 and Anticardiolipin Antibodies in Primary Biliary Cholangitis Patients with Portal Hypertension: A Pilot Retrospective Study

**DOI:** 10.3390/ijms27198461

**Published:** 2026-09-23

**Authors:** Alicja Bauer, Tomasz Rawa, Tomasz Bauer

**Affiliations:** 1 Department of Biochemistry and Molecular Biology, Centre of Postgraduate Medical Education, Marymoncka 99/103, 01-813 Warsaw, Poland; 2 Department of Gastroenterology, Hepatology and Clinical Oncology, Centre of Postgraduate Medical Education, Roentgena 5, 02-781 Warsaw, Poland; tomasz.rawa@nio.gov.pl; 3 Faculty of Chemistry, University of Warsaw, L. Pasteura 1, 02-093 Warsaw, Poland

**Keywords:** primary biliary cholangitis, endothelin-1, anticardiolipin antibodies, liver fibrosis, portal hypertension

## Abstract

Primary biliary cholangitis (PBC) is a chronic autoimmune, cholestatic liver disease leading to fibrosis, cirrhosis, and portal hypertension. Endothelin-1 (ET-1), a potent vasoconstrictor with profibrotic properties, has been implicated in the pathogenesis of liver fibrosis and portal hypertension. Anti-cardiolipin antibodies (aCL) may further contribute to disease progression by promoting thrombotic complications. Our aim was to evaluate serum ET-1 concentrations and anti-cardiolipin antibody levels in PBC patients and their association with portal hypertension. Serum samples from 80 patients with PBC, including patients with portal hypertension, and 25 healthy controls were analyzed. ET-1 concentrations and anti-cardiolipin antibodies were measured using commercially available immunoassays. Elevated serum ET-1 levels were detected in 84% of patients with PBC and portal hypertension and the ET-1 concentrations were also significantly higher in this group compared to the control group. Serum ET-1 levels positively correlated with total bilirubin concentrations and the severity of histological fibrosis. Anti-cardiolipin antibodies were detected in 32% of PBC patients with portal hypertension. Elevated serum ET-1 concentrations and the presence of anti-cardiolipin antibodies are associated with advanced liver disease and portal hypertension in patients with PBC. These biomarkers may have potential value in identifying patients at increased risk of disease progression and worsening liver function.

## 1. Introduction

Primary biliary cholangitis (PBC) is a cholestatic, autoimmune liver disease characterized by the occurrence of anti-mitochondrial (AMA) and antinuclear antibodies in the patients’ sera [1,2,3,4,5,6,7]. The diagnosis of PBC takes into account biochemical markers indicating cholestasis, immunological markers, radiological findings and, of course, the patient’s clinical condition [2,8]. AMA is found in up to 90% of patients with PBC and the main subtype of AMA is anti-M2, directed against pyruvate dehydrogenase complex PDC-E2 [9]. Antinuclear antibodies (ANA) are antibodies against nuclear envelope (NE) proteins, such as anti-gp210 or anti-p62, and also against the intranuclear protein Sp100 [4,10,11,12,13,14,15,16,17,18].

Studies suggest that the pathological progression of PBC is very often associated with inflammation within the portal area [19]. The inflammatory-destructive process leads to progressive fibrosis, cirrhosis and liver failure, and in some cases portal hypertension. The histopathological mechanisms causing portal hypertension in patients with PBC are so far not well understood [20]. In chronic liver injury, hepatic stellate cells located in the perisinusoidal space become active, and their phenotype resembles that of myofibroblasts. Activated stellate cells can begin to produce endothelin-1 [21].

Endothelins (ETs) are a family of three 21-amino-acid peptides: ET-1, ET-2, and ET-3 [21,22,23]. Endothelin-1 (ET-1) is a 21-amino-acid peptide with the chemical formula C_109_H_159_N_25_O_32_ and a molecular weight of 2491.90 g/mol [Figure 1]. It features two essential disulfide bridges. The amino acid sequence of endothelin-1 is H-Cys-Ser-Cys-Ser-Ser-Leu-Met-Asp-Lys-Glu-Cys-Val-Tyr-Phe-Cys-His-Leu-Asp-Ile-Ile-Trp-OH (contains 4 cysteine residues forming 2 critical disulfide bonds). Initially, it is synthesized as a large peptide composed of more than 200 amino acids—preproendothelin-1. Subsequently, an inactive, 38-amino-acid product, Big-endothelin-1, is formed. Big-endothelin-1 under the action of membrane-bound metalloproteases (ECE) releases the mature, active, 21-amino acid ET-1 molecule [Figure 1].

Endothelin-1 binds to two distinct G-protein-coupled cell surface receptors: ET_A_ and ET_B_.

ET_A_ found on vascular smooth muscle cells mediates intense vasoconstriction and cell proliferation and ET_B_ found on endothelial cells typically promotes clearance of ET-1 and the release of vasodilators like nitric oxide [24,25,26].

ET-1, the most potent vasoactive isoform, is widely expressed in endothelial, epithelial, and immune cells [22,23,27,28,29,30,31,32]. It plays a central role in maintaining vascular tone and regulating vascular homeostasis [26,29]. Consequently, early studies focused primarily on its role in the systemic regulation of vascular tone. More recent evidence suggests that ET-1 also contributes to the regulation of blood flow in specific tissues [33,34]. Elevated ET-1 levels have been associated with vasoconstriction, vascular hypertrophy, inflammation, pulmonary fibrosis, and other pathological processes, including cell proliferation and tissue fibrosis [35,36]. A profibrotic role of ET-1 has been demonstrated in patients with idiopathic pulmonary fibrosis (IPF) [31], and several studies have suggested that ET-1 may serve as a potential biomarker for systemic sclerosis-associated interstitial lung disease (SSc-ILD) [30,36,37].

ET-1 plays a particularly interesting role in the liver, where alterations in blood flow contribute to the development and progression of severe complications, including portal hypertension, ascites, and hypoxic liver injury [19,21,36,37,38]. Serum level of endothelin-1 is of high sensitivity and specificity in the diagnosis of hepatocellular carcinoma. ET-1 secretion in HCC is stimulated by many cytokines and growth factors [38]. It appears that ET-1 may be associated with scar tissue formation, which would accelerate liver fibrosis and cirrhosis [39]. We suggest that ET-1 may also play a role in the processes of tissue damage, inflammation, and fibrosis in PBC.

Liver cirrhosis can also be a cause of portal vein thrombosis. The presence of anticardiolipin antibodies (aCL) in the blood disrupts proper coagulation and is associated with an increased predisposition to form blood clots. Elevated levels of anticardiolipin antibodies (aCL) have previously been observed in patients with liver disease. It appears that it may lead to thrombocytopenia and the formation of dangerous blood clots in the liver’s blood vessels [40,41]. Higher concentrations of anticardiolipin antibodies were observed in elderly patients following a stroke [42]. Antiphospholipid antibodies and anticardiolipin antibodies were also investigated in women with recurrent pregnancy loss [43]. High-avidity aCL IgG in PBC may be associated with more advanced liver disease or thrombocytopenia [44,45].

The aim of the study is to determine the concentration of ET-1 and the level of anti-cardiolipin antibodies in the blood serum of patients with portal hypertension in the course of PBC and evaluate the potential utility of these parameters as diagnostic biomarkers of progression and complications.

## 2. Results

### 2.1. Clinical, Histological and Laboratory Features of PBC

Table 1 presents clinical, histological, and laboratory data of patients with PBC.

The group of patients with PBC consisted of 76 women and four men. The mean age at the time of PBC diagnosis was 52 years. Elevated total bilirubin levels were observed in over 60% of the patients studied (normal value < 1.2 mg/dL). Increased AP and γ-GT activity was present in over 70% of patients, while AST and ALT activity was also elevated in over 65% of patients. The normal value for AST was <40 U/L; ALT < 40 U/L; AP < 115 U/mg/dL; -GT < 50 U/L; albumin 3.5–5.5 g/dL, -globulin < 3 g/dL. AMA M2, specific autoantibodies for PBC, were noticed in 88% of patients’ sera.

All patients underwent a liver biopsy. In 50 cases (63%), minor histopathological changes were observed (stage I or II according to the Ludwig classification), while in 25 cases (31%), the biopsy revealed advanced fibrosis (stage III or IV according to the Ludwig classification). Portal hypertension was diagnosed in 25% of patients.

### 2.2. Endothelin-1 in the Serum of Patients with PBC

Elevated levels of ET-1 were detected in the sera of 84% (21/25) of patients with PBC and portal hypertension, while in the group of PBC patients without portal hypertension they were detected only in 40% (22/55), *p* < 0.001 [Figure 2].

In the control group, elevated ET-1 levels were detected in only two subjects. The distribution of ET-1 in PBC patients with and without portal hypertension and in the healthy control group is shown in Figure 3.

A significantly higher concentration of ET-1 was observed in the group of PBC patients with portal hypertension compared to PBC patients without symptoms of portal hypertension, 6.9 ± 4.3 pg/mL vs. 2.5 ± 2.9 pg/mL, *p* < 0.001 [Figure 4].

The ET-1 level correlated with serum total bilirubin (*p* < 0.05) [Figure 5] and also correlated with the severity of histologic fibrosis [Table 2].

There is significant statistical difference between patients with portal hypertension compared to PBC patients without portal hypertension in this group with advanced fibrosis. The concentration of ET-1 was significantly higher, about two times.

Receiver-operating characteristic curve analysis for serological detection of ET-1 in PBC patients with portal hypertension is presented in Figure 6.

The calculated AUC value (Area Under the Curve) of 0.86 for the group of PBC patients with portal hypertension is excellent, indicating that this model has a strong ability to separate the positive and negative serum. The specificity of serum ET-1 for PBC was 79%.

### 2.3. Anticardiolipin Antibodies in the Sera of PBC Patients

Anticardiolipin antibodies were detected in 18% (14/80) of all PBC patients tested. The vast majority of patients with a positive result for aCL were patients diagnosed with portal hypertension, 32% (8/25), vs. 12% (7/55) for the group without portal hypertension, *p* = 0.009 [Figure 7].

In the healthy control group, aCL antibodies were observed in one patient. The specificity of aCL antibodies for diagnosis of PBC with portal hypertension was 74%. The presence of anti-cardiolipin antibodies and elevated ET-1 levels together was observed significantly more often in patients with portal hypertension [Figure 8].

The incidence of aCL and increased ET-1 concentration in the sera of patients with PBC was also associated with more advanced cirrhosis of the liver OR (95% CI)—13.5 (1.5–122.8), *p* = 0.021.

## 3. Discussion

Portal hypertension is a major complication of cirrhosis, and its consequences, including ascites, esophageal varices, and hepatic encephalopathy, lead to substantial morbidity and mortality. ET-1 is one of the most potent endogenous vasoconstrictors and it is implicated in the pathogenesis of portal hypertension through its effects on hepatic sinusoidal resistance and vascular remodelling. In addition to its vasoactive properties, ET-1 stimulates hepatic stellate cell activation, extracellular matrix deposition, and collagen synthesis, supporting its role as a profibrotic mediator. Activated stellate cells begin to produce endothelin-1, which consequently leads to an imbalance between profibrotic and antifibrotic factors. Increased ET-1 production by endothelial cells, hepatic stellate cells, and cholangiocytes may contribute to enhanced intrahepatic vascular resistance, thereby promoting the development of portal hypertension. It has been shown that ET-1 can contribute to the activation of hepatic stellate cells and an increase in sinusoidal tone [21,46]. This may be one of the key mechanisms underlying the rise in portal pressure [21,46,47,48,49]. ET-1 narrows the tiny blood vessels (sinusoids) inside the liver, which raises blood pressure in the portal vein. A damaged liver may produce increased amounts of ET-1. This leads to the contraction of stellate cells and, consequently, the stiffening of liver tissue.

Damage to the sinusoidal endothelium can lead to secondary liver injury. Parenchymal cells would be exposed to toxic substances that, under normal conditions, should be removed by the cells lining the sinusoids cells [47]. The deterioration of perfusion regulation within the sinusoids is specifically related to increased ET-1 secretion and decreased production of vasodilatory mediators such as nitric oxide (NO). The presence of anticardiolipin antibodies may be associated with hepatic vascular complications, including portal hypertension [50,51].

While limited data exist in the literature regarding the relationship between ET-1 and portal hypertension, our aim was not only to link ET-1 to portal hypertension but also to investigate the associations among anticardiolipin antibodies, ET-1, and portal hypertension.

Our questions were:

Is ET-1 involved in liver progression and can it be a marker of fibrosis and portal hypertension?

What is the diagnostic role of the presence of anti-cardiolipin antibodies in the serum of PBC patients?

In our study, patients with PBC and portal hypertension had significantly more frequent elevations in ET-1 levels than patients without diagnosed portal hypertension. Higher ET-1 levels correlate with increased serum total bilirubin and greater histological fibrosis, indicating more advanced liver damage. We suggest that ET-1 may act on hepatic stellate cells and endothelial cells. This induces cell contraction and increases vascular tone. Concurrently, this process can lead to the accumulation of extracellular matrix, ultimately resulting in fibrosis and portal hypertension. A study by Ratti et al. also confirmed that serum levels of endothelin-1 can be an early predictor of postoperative liver failure after liver resection [52]. Our study showed that there was a statistically significant increase in ET-1 levels in the liver cirrhosis group in comparison to the normal control group. These results are consistent with previous studies indicating increased circulating ET-1 concentrations in patients with advanced chronic liver disease and portal hypertension [49,52]. The observed association between ET-1 and the degree of histological fibrosis in our study is consistent with these biological mechanisms and suggests that ET-1 may reflect both fibrogenesis and vascular dysfunction in PBC. ET-1 may be associated with endothelial dysfunction and increased vascular resistance in the portal circulation. Elevated levels of endothelin-1 in the liver and blood contribute to disease progression and vascular changes.

Another important result was the higher incidence of anticardiolipin antibodies (aCL) among patients with portal hypertension and advanced cirrhosis. The presence of anticardiolipin antibodies can show an increased risk of thrombotic complications, both venous and arterial. Testing for these antibodies is indicated when thrombotic symptoms or antiphospholipid syndrome are suspected; however, the presence of aCL antibodies may also indicate immune-mediated vascular abnormalities. Their presence has also been reported in the course of chronic cholestatic liver diseases, including autoimmune liver disorders [41,53]. Liver cirrhosis can also be a cause of portal vein thrombosis.

The simultaneous presence of elevated ET-1 levels and aCL was significantly more common in our patients with portal hypertension than in those without this complication. The obtained results demonstrate that endothelin-1 (ET-1) and anticardiolipin antibodies (aCL) are related to a more progressive stage of the disease in patients with primary biliary cholangitis (PBC), particularly in those with portal hypertension. It appears that in the course of PBC, the presence of aCL antibodies may reflect persistent immune system activation, but it may also be associated with thrombotic disease. Microcirculation significantly influences complex liver functions related to the metabolism, biosynthesis, clearance, and the body’s defence mechanisms. Anti-cardiolipin antibodies may contribute to endothelial dysfunction, microcirculatory damage, and a prothrombotic state. These factors may be associated with intrahepatic vascular abnormalities and promote the progression of portal hypertension. Our results suggest that endothelial dysfunction and autoimmune-mediated vascular damage may coexist and exacerbate disease progression. ET-1 is associated with increased vasoconstrictive activity and profibrotic effects, whereas aCL antibodies may identify patients with additional vascular and immunological abnormalities. Together, these biomarkers may therefore provide complementary information regarding the severity of liver disease and the risk of portal hypertension.

There is almost no research regarding the assessment of ET-1 in the course of PBC, only a few related to other liver diseases, and the association of this molecule with anticardiolipin antibodies has not been analyzed [53]. Elbadry et al. investigated the presence of portal vein invasion in hepatocellular carcinoma (HCC) and found no statistically significant association between endothelin-1 levels and portal vein thrombosis in the course of HCC [40]. The fact that no association was found between endothelin-1 concentration and portal vein thrombosis in HCC does not rule out the possibility of such a relationship in PBC, particularly given the coexistence of elevated ET-1 levels and aCL antibodies with advanced disease progression.

When investigating the link between ET-1 and thrombosis, one might consider a pro-coagulant effect, as ET-1 induces the release of von Willebrand factor (vWF) from cells, thereby promoting platelet aggregation and clot formation. This opens the door for future research, specifically studies focusing on PBC patients with portal vein thrombosis. As this is a relatively rare complication, assembling a sufficiently large patient cohort is challenging; therefore, multicenter studies would be advisable. In our study group of 80 patients, only one presented with portal vein thrombosis. Although this patient exhibited both aCL antibodies and elevated endothelin-1 levels, these findings were excluded from the main analysis and are suitable only for a single case report.

It can be hypothesized that the coexistence of increased endothelin-1 (ET-1) activity and the presence of anticardiolipin antibodies contributes to the pathogenesis of portal hypertension in PBC. Through its vasoconstrictive and profibrotic actions, ET-1 may enhance the contraction of activated hepatic stellate cells and promote increased extracellular matrix deposition, potentially leading to elevated intrahepatic vascular resistance. Anticardiolipin antibodies may also promote endothelial and coagulation system activation, potentially resulting in microcirculatory disturbances and microthrombus formation. Evidence also suggests that anticardiolipin antibodies can stimulate ET-1 expression, implying a potential synergistic interaction between vasoconstrictive and prothrombotic mechanisms. It has been experimentally demonstrated that aCLs significantly enhance prepro-ET-1 mRNA expression and increase the secretion of mature ET-1 by the endothelium [54,55]. Both aCL and ET-1 stimulate the overexpression of adhesion molecules on the endothelial surface. This facilitates leukocyte recruitment and adhesion, initiating local inflammation. Complement system activation by aCL can stimulate neutrophils to release neutrophil extracellular traps (NETs). aCL antibodies strongly activate monocytes and endothelial cells to express tissue factor (TF), the primary activator of the coagulation cascade [56]. ET-1 independently upregulates TF expression in macrophages and the vessel wall, acting as a direct enhancer of the prothrombotic effects of aCL. The potent and sustained vasoconstrictive action of ET-1 narrows the vessel lumen and restricts blood flow. Combined with aCL-induced microthrombi, this leads to chronic tissue hypoxia. Hypoxia and the presence of inflammatory cytokines—the secretion of which can be enhanced by ET-1—trigger the differentiation of fibroblasts into myofibroblasts. Myofibroblasts, in turn, produce massive amounts of collagen and extracellular matrix proteins. It appears that in PBC, a potential synergism between the profibrotic and vasoconstrictive effects of ET-1 and the prothrombotic and immunological actions of aCL could drive the progression of hepatic vascular lesions, increase intrahepatic resistance, and lead to the development or worsening of portal hypertension. The combination of ET-1’s vasoconstrictive and profibrotic profile with the prothrombotic and immunological effects of aCL results in accelerated endothelial dysfunction and vascular remodelling. However, this hypothesis requires confirmation through clinical and mechanistic studies.

Therefore, the present study extends current knowledge by suggesting that both ET-1 and aCL are associated with advanced disease manifestations in PBC.

In clinical cardiopulmonary practice, therapeutic interventions that aim to inhibit ET-1 using receptor blockers or to stimulate the production of nitric oxide, which has a vasodilating effect, are employed [57,58,59]. Possibly similar solutions could also be applied in portal hypertension.

Several limitations should be acknowledged. The observed correlation between ET-1 concentration and the analyzed parameters does not allow for a definitive conclusion regarding a cause-and-effect relationship, nor does it confirm a direct role for ET-1 in cell injury and the development of fibrosis. Therefore, these results should be interpreted as indicating a potential association between ET-1 and the processes occurring during the course of PBC, rather than as evidence of a direct pathogenetic role. However, the observed associations may suggest a potential role for ET-1 in the pathogenesis of these processes and justify the need for further research. Indeed, experimental studies using animal models and studies involving the assessment of histological material from patients appear particularly important, as they would allow for the direct verification of the relationship between ET-1 expression, cell injury, and the severity of fibrosis. But, in this study, we aimed to focus primarily on the role of ET-1 as a prognostic marker with potential diagnostic utility. It should be emphasized that demonstrating an association between ET-1 concentration and selected biochemical parameters or markers of injury constitutes a significant clinical observation, indicating the rationale for further research into the role of this peptide in the pathogenesis of PBC.

The work included a relatively small number of patients with portal hypertension, limiting statistical power. Portal hypertension was evaluated at a single time point, and longitudinal studies are required to determine whether these biomarkers can predict disease progression or response to treatment. Portal vein pressure is associated with damage to sinusoidal endothelial cells and may rise following extensive liver resection; future prospective studies could help determine whether there is a correlation between changes in portal pressure during liver resection and ET-1 levels, as well as rule out any potential link between systemic hemodynamic changes and ET-1.

Another limitation of the study is the uneven gender distribution within the analyzed groups. The observed gender structure was not solely a result of the sampling method but largely reflected the actual demographic profile of the PBC patient population; the predominance of women among PBC patients aligns with the epidemiological characteristics of the disease, which occurs much more frequently in women. At the same time, the small number of men in both groups precludes a reliable analysis of results stratified by gender and limits the generalizability of the findings to the male PBC population. In the group of 80 PBC patients, women accounted for 95% of the subjects (76 women) and men for 5% (four individuals). A similar trend was observed in the control group of 25 individuals, where women constituted 84% (21 women) and men 16% (four individuals). A clear female predominance was evident in both analyzed groups, which reduces the likelihood that differences between the PBC group and the control group were significantly attributable to differing gender structures. Consequently, the gender structure should be considered a study limitation; however, the impact of this factor on the group comparison is partially mitigated by the fact that the gender imbalance followed a similar pattern in both the study and control groups. The results obtained should be interpreted primarily with reference to the population of women with PBC. Further research is required to confirm the observed associations in larger cohorts that include a sufficient number of men.

In conclusion, elevated serum ET-1 concentrations and the presence of anticardiolipin antibodies are associated with portal hypertension, advanced fibrosis, and cirrhosis in patients with PBC. These findings support the hypothesis that endothelial dysfunction and immune-mediated vascular injury contribute to disease progression. Further prospective studies are warranted to determine whether ET-1 and aCL may serve as clinically useful biomarkers for risk stratification and monitoring of patients with PBC.

## 4. Materials and Methods

### 4.1. Patients

Serum samples were composed from 80 PBC patients (76 women, four men; median age: 52; range: 33–71 years), detected at the Centre of Postgraduate Medical Education (Warsaw, Poland). The diagnosis of PBC was recognized using generally accepted criteria, according to the practical guidelines of the European Association for the Study of the Liver (EASL) for PBC [60]. A biopsy was performed in all subjects. In AMA-negative PBC patients, diagnosis was supported by antinuclear antibody positivity or by liver biopsy.

In patients with suspected portal hypertension or esophageal varices, the following procedures were performed: Doppler ultrasound of the upper abdomen (blood flow patterns indicating slowed flow suggested portal hypertension), gastrointestinal endoscopy, and computed tomography (which provided detailed cross-sectional images of the liver and surrounding blood vessels; an enlarged spleen or new, dilated, or tortuous veins referred to as collateral circulation or varices suggested the presence of portal hypertension). Liver elastography was also performed in some patients, and hepatic venous pressure gradient (HVPG) measurement in others. Portal hypertension was defined based on an HVPG value > 5 mmHg.

We excluded patients with serum levels positive for the hepatitis B surface antigen (HBsAg), anti-hepatitis A (IgM) and hepatitis C virus, patients with alcoholism and AIH (autoimmune hepatitis)/PBC overlap syndrome. In most patients, the diagnosis was made within one year after the onset of symptoms. The control group consisted of 25 healthy adult blood donors (21 females, 4 males; median age: 45; age range: 25–55 years).

The study protocol was conducted in agreement with the ethical guidelines of the Declaration of Helsinki and was accepted by the Ethical Committee of the Centre of Postgraduate Medical Education, Warsaw, Poland (approval number 71/PB/2019, 12 June 2019).

### 4.2. Detection of ET-1

The ET-1 concentration was assessed using a commercially available ELISA kit (DRG^®^ Instruments GmbH, Marburg, Germany), according to the manufacturer’s instructions. Intra-assay and inter-assay performances were 8.1% and 9.9%, respectively. ET-1 concentrations > 2.8 pg/mL were considered positive. To determine the cut-off value, a reference interval method (mean ± 2 SD) was applied to a control group of healthy individuals. The mean and standard deviation (SD) of the measured values were calculated. The upper limit of normal was set at the mean ± 2 SD, a range that encompasses approximately 95% of the healthy group.

### 4.3. Detection of Anticardiolipin Antibodies

Anticardiolipin IgG antibodies level was assessed using a commercially available ELISA kit (Wuhan Fine Biotech Co., Ltd., Wuhan, China), according to the manufacturer’s instructions. Intra-assay and inter-assay performances were 5.3% and 5.9%, respectively. Anticardiolipin levels > 14 ng/mL were considered positive. To determine the cut-off value, a reference interval method (mean ± 2 SD) was applied to a control group of healthy individuals. The mean and standard deviation (SD) of the measured values were calculated. The upper limit of normal was set at the mean ± 2 SD, a range that encompasses approximately 95% of the healthy group.

### 4.4. Statistical Analysis

Prevalence rates were compared between groups using the chi-square test and Fisher’s exact test. Continuous variables are presented as mean ± standard deviation (SD), while categorical variables are presented as numbers and percentages. Student’s *t*-test was used to compare continuous variables between groups. A *p* value < 0.05 was considered statistically significant. All statistical analyses were performed using MedCalc for Windows, version 7.4.1.0 (MedCalc Software, Mariakerke, Belgium).

## Figures and Tables

**Figure 1:**
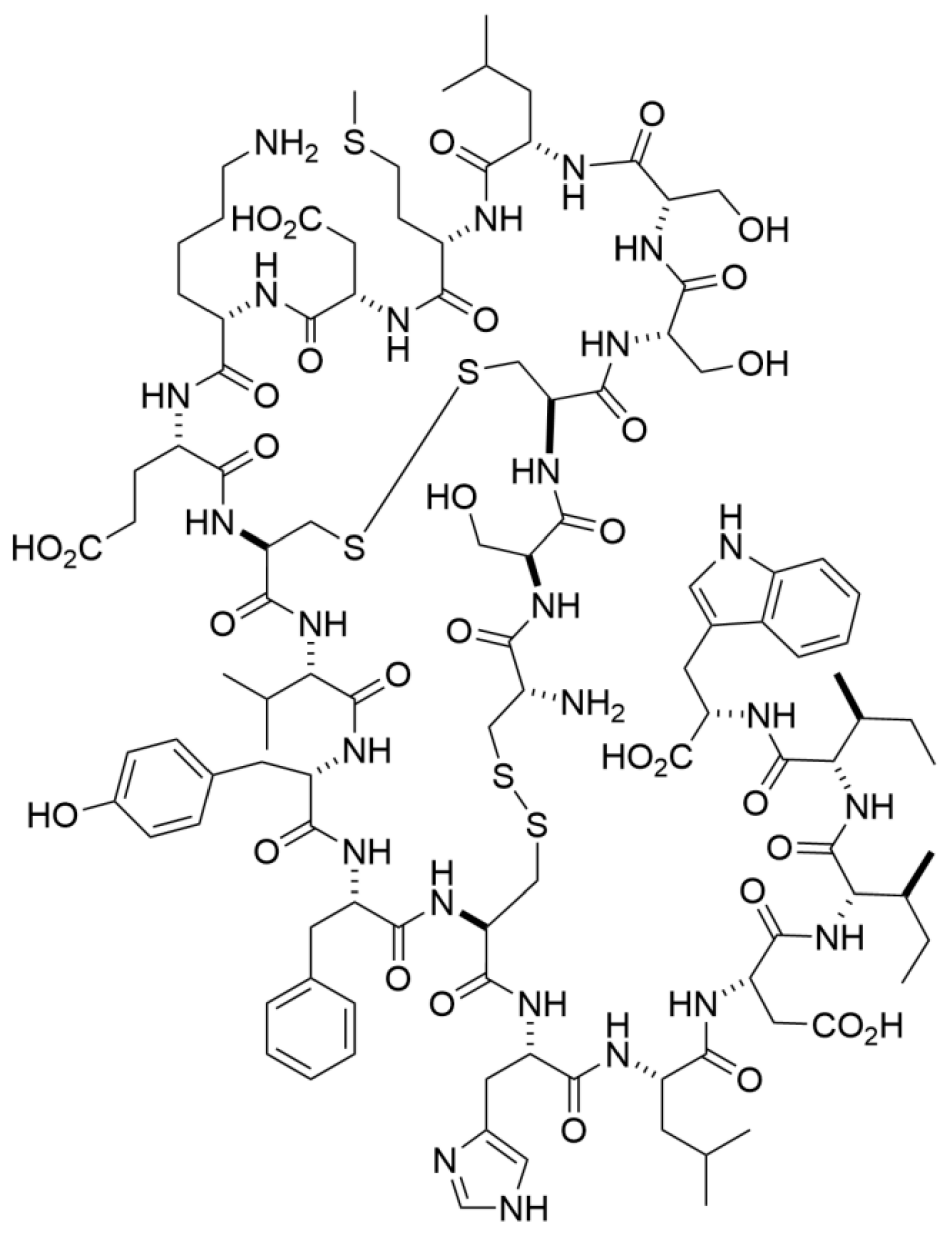
Endothelin-1.

**Figure 2:**
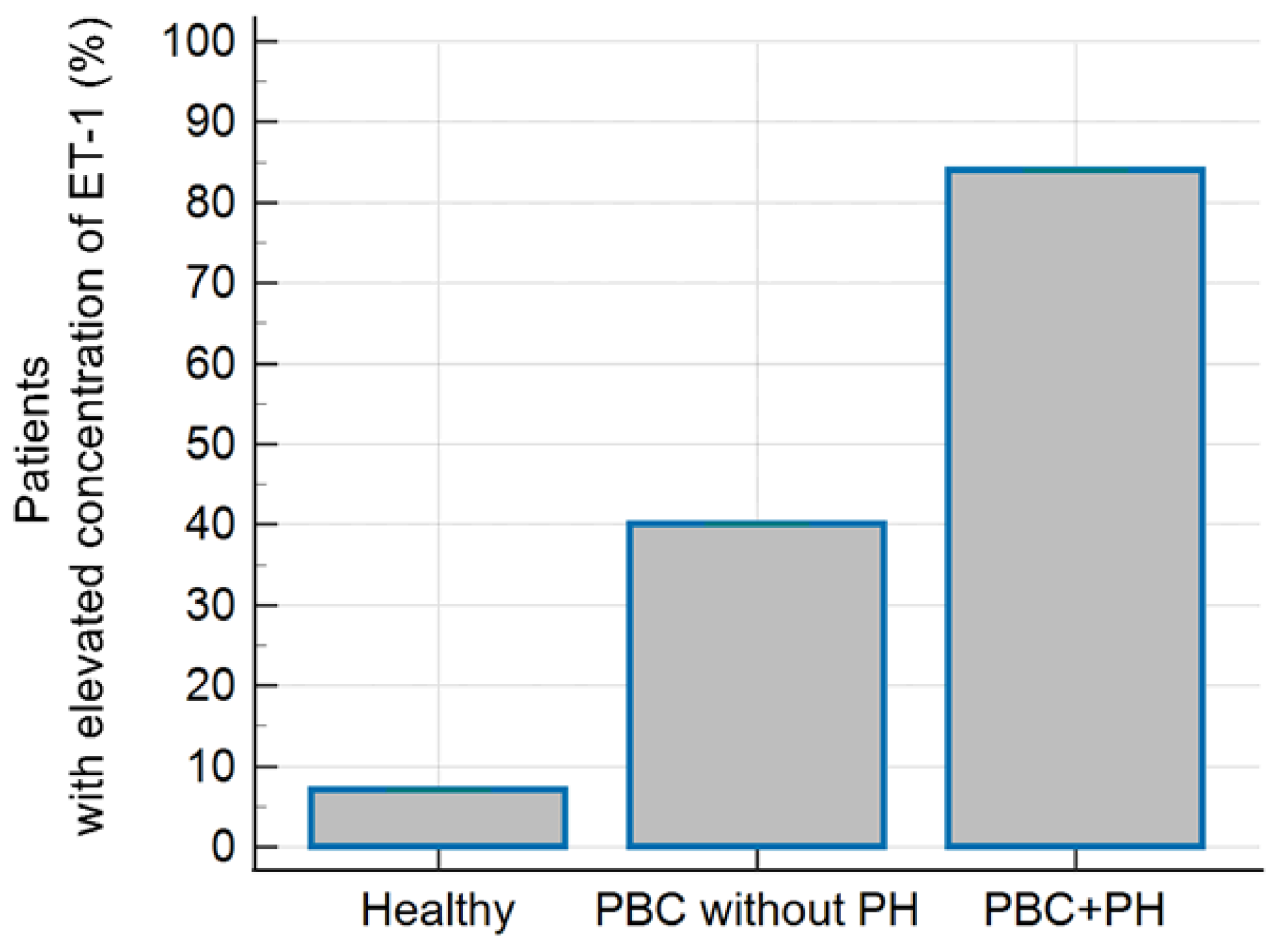
Occurrence of endothelin-1 (ET-1) in the sera of patients with primary biliary cholangitis (PBC) and healthy control; PH—portal hypertension.

**Figure 3:**
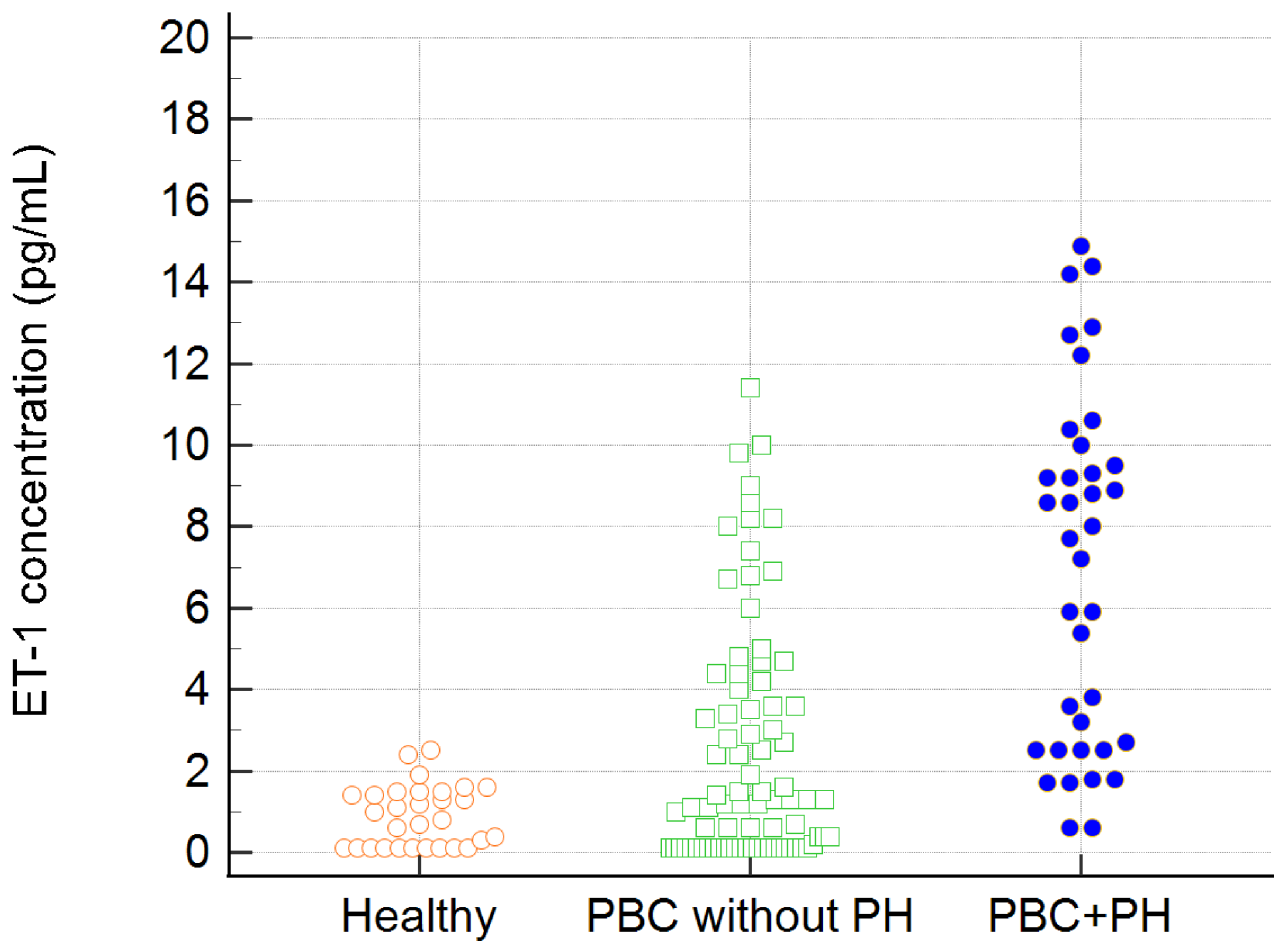
The distribution of endothelin-1 (ET-1) in each of the tested groups. PBC—primary biliary cholangitis, PH—portal hypertension.

**Figure 4:**
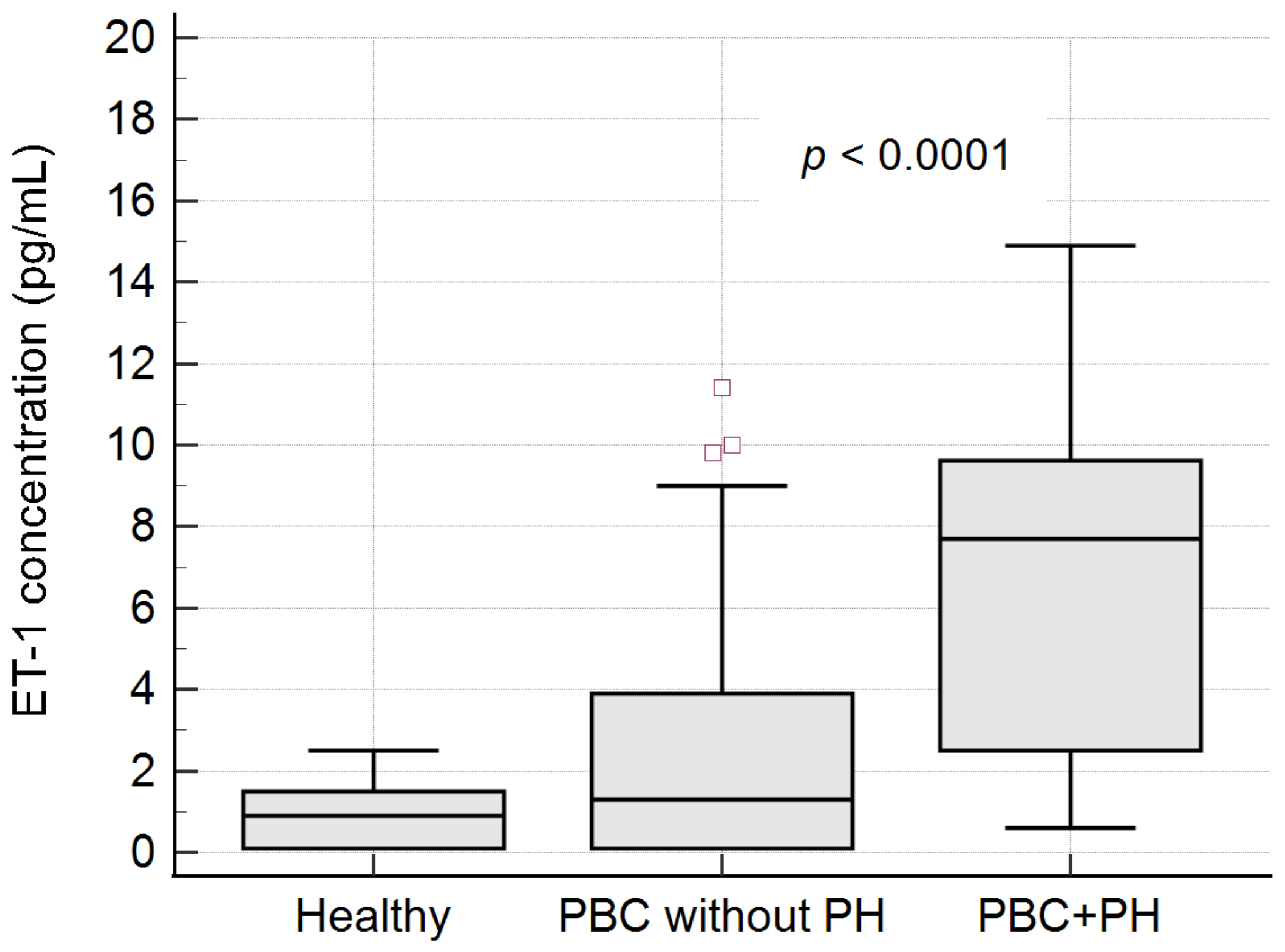
The mean concentration of endothelin-1 (ET-1) in the sera of patients with primary biliary cholangitis (PBC) healthy controls. PH—portal hypertension.

**Figure 5:**
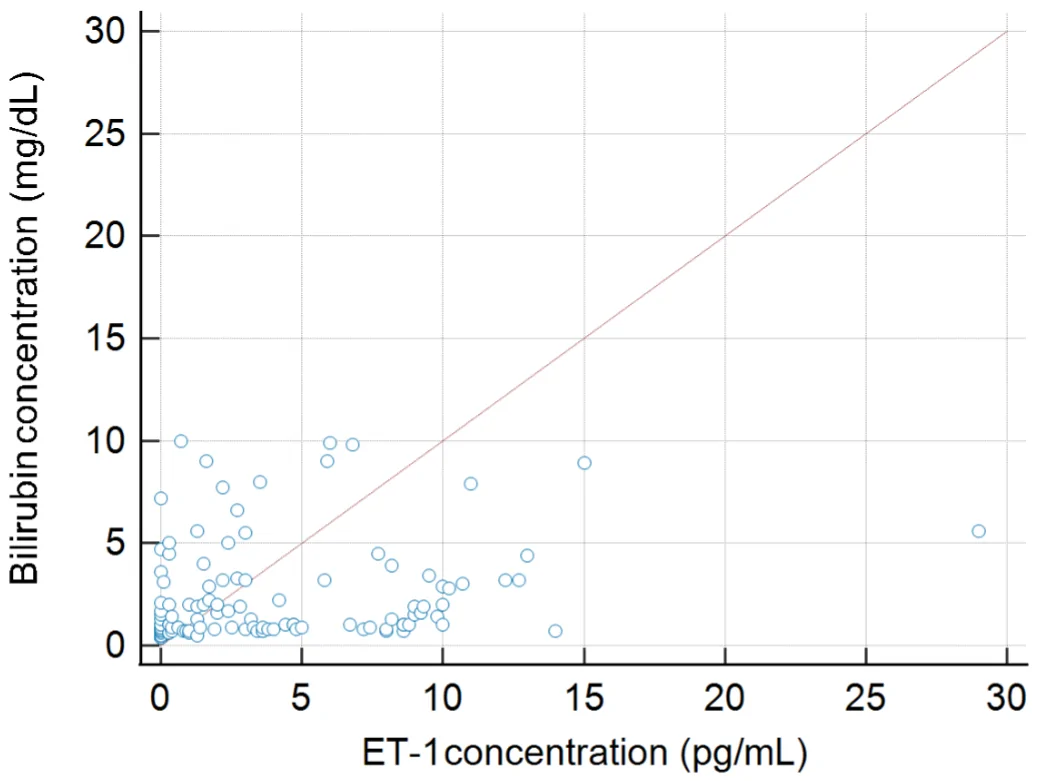
Correlation between serum endothelin-1 (ET-1) concentration and bilirubin levels in patients with primary biliary cholangitis (PBC).

**Figure 6:**
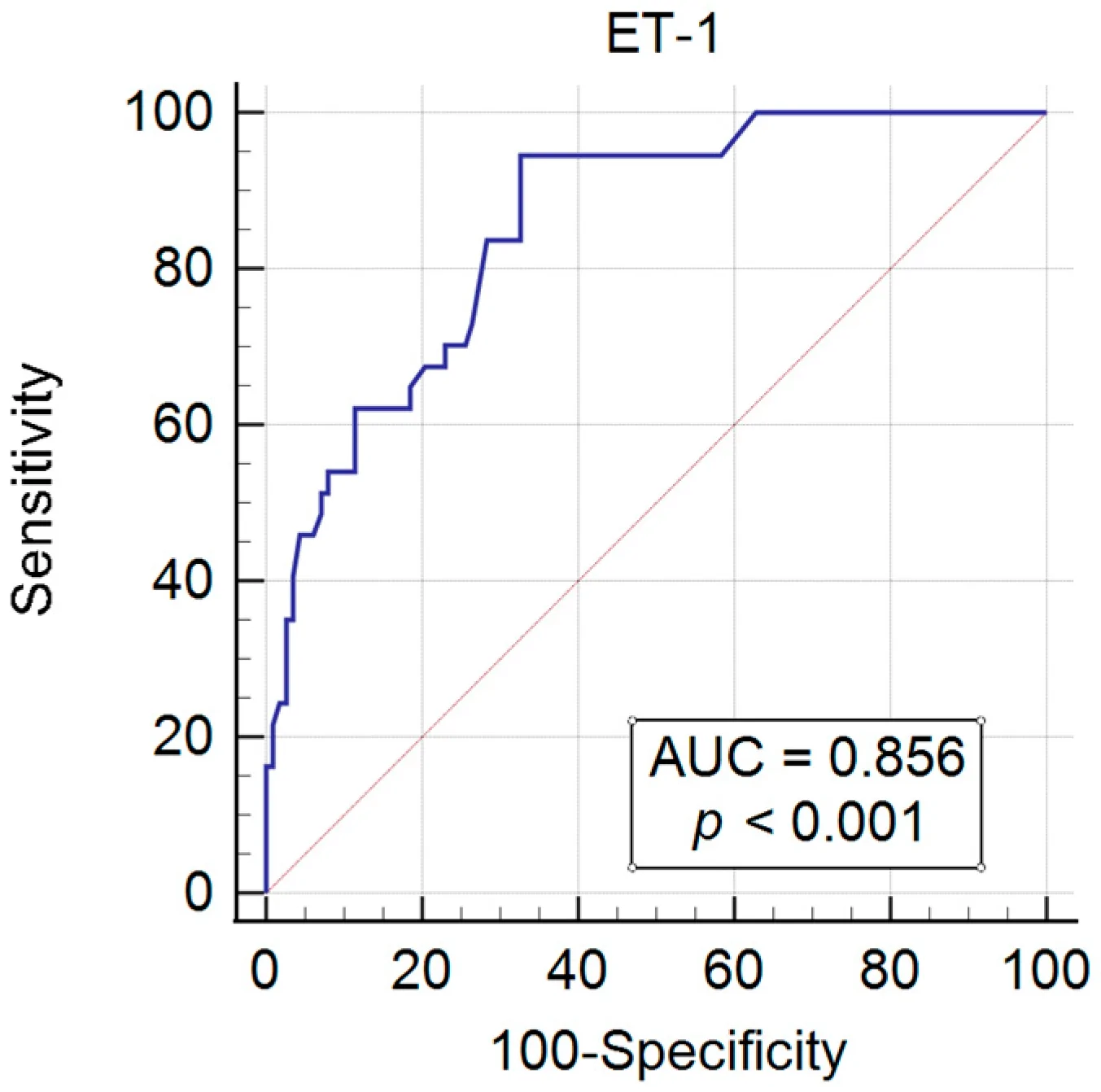
Receiver-operating characteristic curve (ROC) analysis for serological detection of endothelin-1 (ET-1) in PBC patients with portal hypertension.

**Figure 7:**
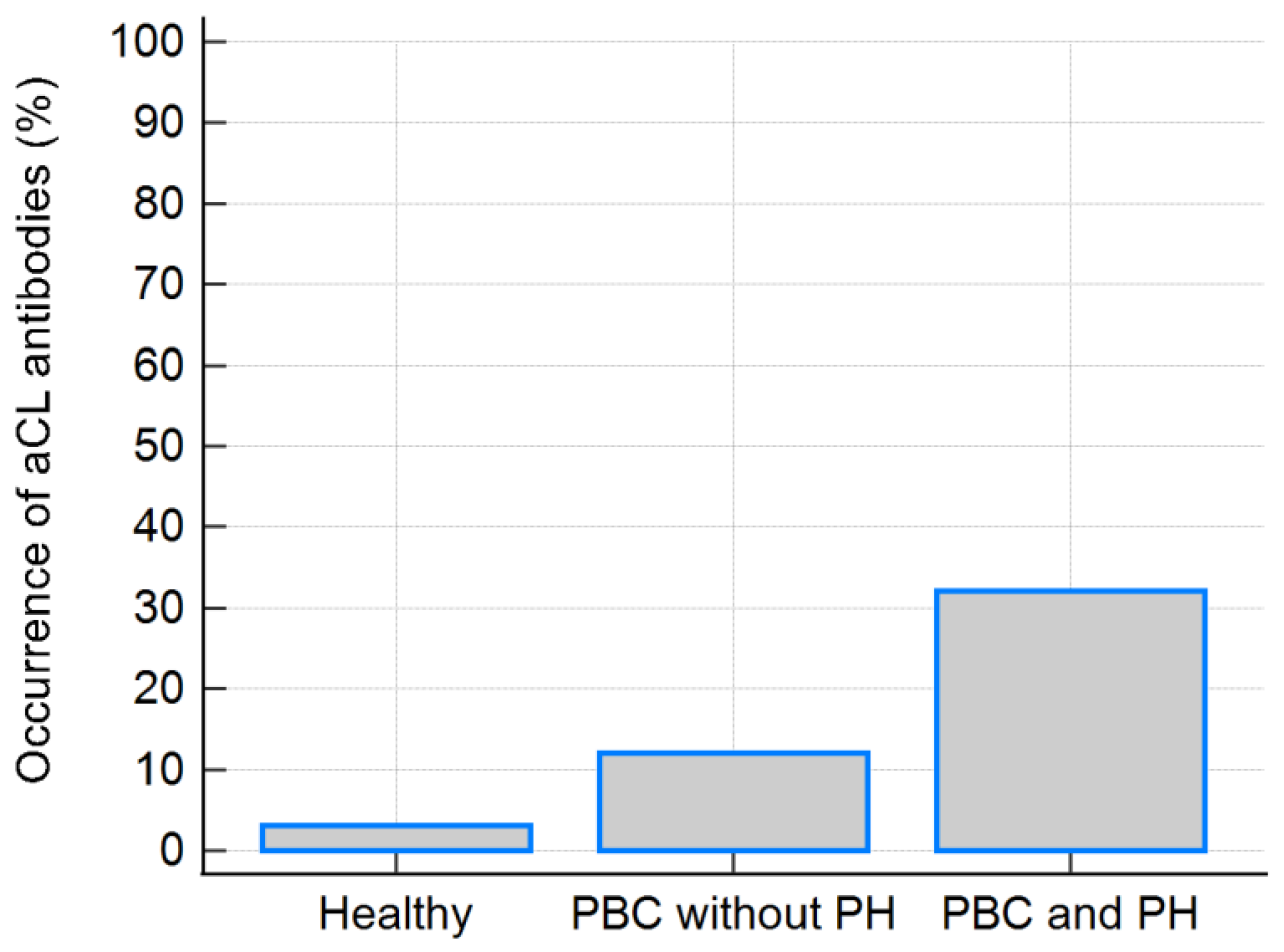
Occurrence of anticardiolipin antibodies (aCL) in patients with primary biliary cholangitis (PBC) and healthy controls; PH—portal hypertension.

**Figure 8:**
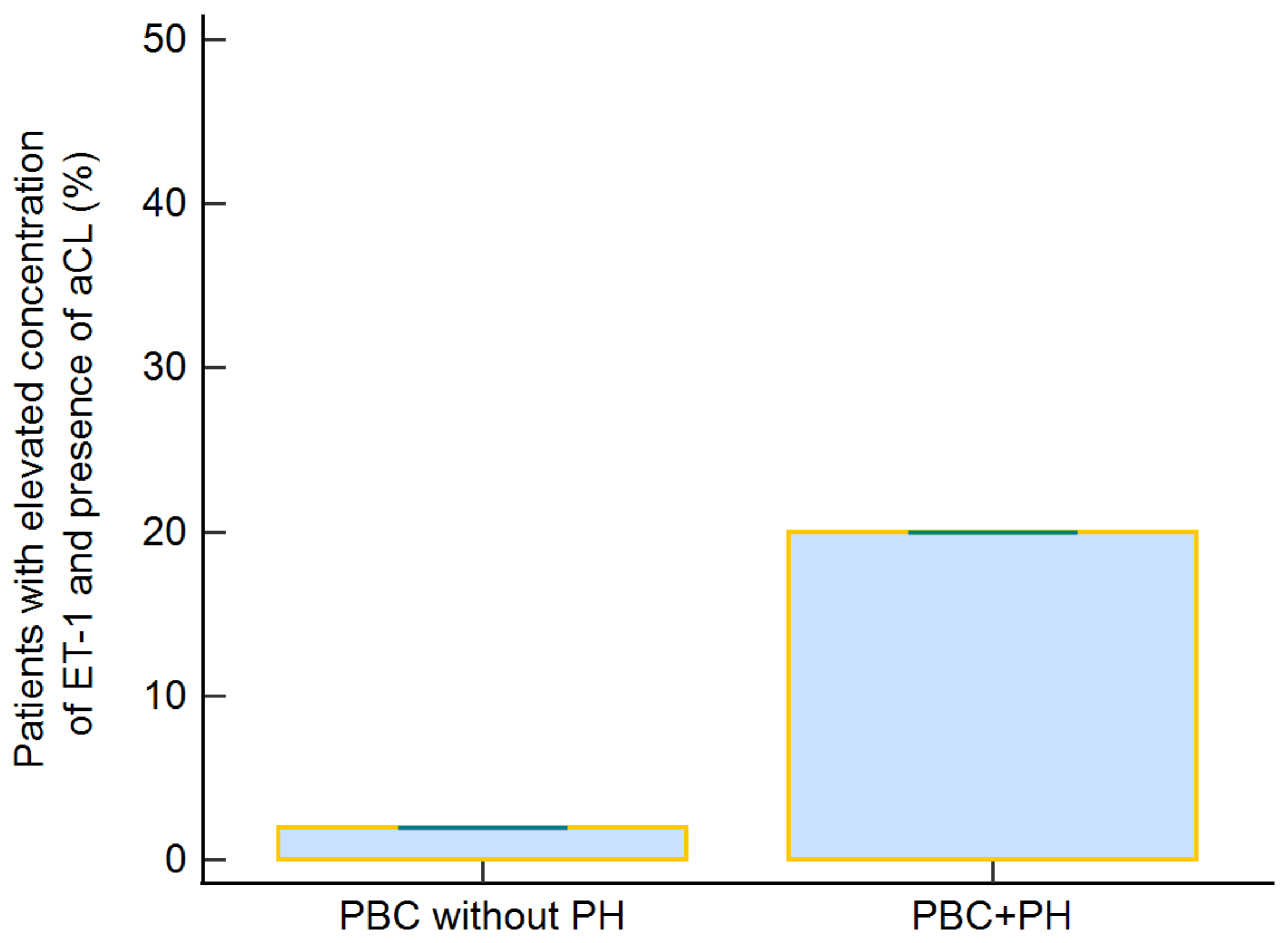
Presence of anticardiolipin antibodies (aCL) and increased endothelin-1 (ET-1) concentration in the sera of patients with primary biliary cholangitis with and without portal hypertension; PH—portal hypertension.

**Table 1:** The demographic, biochemical, immunological and histological features of PBC patients.

	Primary Biliary Cholangitis (PBC) Patients (n = 80)
Age, years	52 (33–71)
Females/males	76/4
Bilirubin (Total), mg/dL	1.8 (1.6)
AST, U/L	76.0 (51.1)
ALT, U/L	82.1 (69.2)
AP, U/L	375.5 (344.1)
γ-GT, U/L	258.4 (241.2)
Albumin (g/dL)	3.6 (0.9)
AMA M2	70 (88%)
Early histological stage (I/II)	50 (63%)
Advanced histological stage (III/IV)	25 (31%)
Liver tissue without changes	5 (6%)
Patients with portal hipertention	25 (31%)

Data are presented as mean (SD). Abbreviations: γ-GT, γ- glutamyl transpeptidase; ALT, alanine aminotransferase; AP, alkaline phosphatase; AST, aspartate aminotransferase. Normal value: bilirubin < 1.2 mg/dL; AST < 40 U/L; ALT < 40 U/L; AP < 115 U/mg/dL; γ-GT < 50 U/L; albumin 3.5–5.5 g/dL, γ-globulin < 3 g/dL. Conversion factors to SI units are as follows: for bilirubin, 17.1; for AST, ALT, AP and γ-GT, 0.0167.

**Table 2:** Endothelin-1 concentration and liver fibrosis in PBC patients.

Liver Fibrosis (Ludwig’s Classification)	PBC Without Portal Hypertension		PBC with Portal Hypertension		*p* Value
	Patients (55)	ET-1 Concentration (pg/mL)	Patients (25)	ET-1 Concentration (pg/mL)	
“0”	5	2.0 ± 2.2	0	-	-
“1”	18	4.0 ± 0.9	7	3.4 ± 2.4	n.s
“2”	21	5.6 ± 2.5	6	6.0 ± 2.7	n.s
“3”	6	6.5 ± 3.4	2	13.9 ± 2.4	0.03
“4”	5	7.7 ± 2.3	10	16.3 ± 5.6	0.01

ET-1—endothelin 1, PBC—primary biliary cholangitis, n.s.—not significant.

## Data Availability

The original contributions presented in this study are included in the article. Further inquiries can be directed to the corresponding author.
